# Concomitance of pulmonary spindle cell carcinoma and sclerosing pneumocytoma in a woman: A case report

**DOI:** 10.1097/MD.0000000000018416

**Published:** 2019-12-20

**Authors:** Xu LuLu, Shu Jian

**Affiliations:** Department of Radiology, The Affiliated Hospital of Southwest Medical University, Luzhou, China.

**Keywords:** computed tomography, lung, sarcomatoid carcinoma, sclerosing hemangioma, sclerosing pneumocytoma, spindle cell carcinoma

## Abstract

**Rationale:**

Pulmonary spindle cell carcinoma (PSCC) is a rare subset of pulmonary sarcomatoid carcinoma. PSCC is aggressive and has a poor prognosis. Pulmonary sclerosing pneumocytoma (PSP) is an asymptomatic slow-growing benign tumor, which usually occurs in middle-aged women.

**Patient concerns:**

Herein, we report a case of solitary PSCC, occurring concomitantly with PSP in a 74-year-old woman. The patient visited our institution with productive purulent cough, dyspnea after activity, and hemoptysis. Enhanced computed tomography revealed an inhomogeneous enhanced mass with central low-attenuation in the right upper lobe (RUL). The mass located in the right lower lobe (RLL) exhibited homogeneous enhancement.

**Diagnosis:**

These lesions were subsequently diagnosed as PSCC in the RUL and PSP in the RLL, following postoperative pathological examination.

**Interventions:**

We performed lobectomy for the RUL and wedge resection for the RLL in one procedure.

**Outcomes:**

The patient did not experience complications after surgery. No radiological evidence of recurrence was observed on follow-up computed tomography performed within 7 months after the procedure.

**Lessons:**

This case fully reflects the importance of the differential preoperative diagnosis of benign and malignant solitary pulmonary nodules. However, a rare and aggressive malignant tumor may have imaging features typical of a lung abscess, which should be treated carefully.

## 1 Introduction

Pulmonary sarcomatoid carcinoma (PSC), a poorly differentiated non-small-cell lung cancer (NSCLC), has 5 rare subtypes and accounts for 0.5% of all lung cancers.^[1]^ The histopathological composition of PSC includes malignant epithelial and sarcomatous elements. According to the current World Health Organization (WHO) criteria, a diagnosis of PSC can be made, if the areas of sarcomatoid change constitute >10% of the tumor.^[2]^ Even after surgery, PSC tends to have a poor prognosis.^[3]^ Therefore, these findings are important for the preoperative diagnosis of PSC.

Pulmonary sclerosing pneumocytoma (PSP) is a rare benign epithelial tumor, which was previously known as “pulmonary sclerosing hemangioma.” It is thought to originate from the primitive respiratory epithelium.^[4]^ It usually occurs in non-smoking middle-aged Asian women. There are no typical clinical symptoms of PSP. It is found incidentally on chest radiography or computed tomography (CT).^[5]^

Herein, we report 2 rare tumors occurring synchronously, a solitary PSC and a PSP, in the right lobe of the same lung.

## 2 Case presentation

A 74-year-old woman presented to our hospital with productive purulent cough for 3 months, dyspnea after activity, and hemoptysis for 1 week. Her medical history included a lump in the lungs, which was discovered 20 years earlier, apart from which, her medical history was completely uneventful. Unenhanced CT revealed 2 separate masses located in the right upper lobe (RUL) and lower lobe (RLL) (Figs. 1 and 2). The peripheral mass located in the RUL showed a central low-attenuation area, marginal irregularity, and a calcification spot (Fig. 1 A and B). The peripheral mass located in the RLL appeared well-defined and round, with multiple nodular calcifications (Fig. 2 A and B). Percutaneous needle biopsy was performed for the mass located in the RUL. Subsequently, contrast-enhanced CT revealed a small intratumoral cavity with an inhomogeneous enhanced mass with central low-attenuation in the RUL (Fig. 1 C and D). Homogeneous enhancement was observed in the mass located in the RLL (Fig. 2 C and D). There were no enlarged lymph nodes in the mediastinum. Laboratory examination revealed neutrophilia (75% neutrophils) and high levels of CA125. Finally, the surgeon decided to perform lobectomy of the RUL and wedge resection of the RLL in a single procedure. During surgery, the mass located in the RUL, which measured 5 cm × 4 cm × 4 cm, appeared gray-white and was poorly circumscribed. Pathological examination established that the mass in the RUL was composed of neoplastic spindle-shaped cells and some polygonal cells with nest-like distribution, surrounded by multinucleated giant cells (Fig. 1 E). Immunohistochemical staining showed that the malignant cells were positive for cytokeratin (CK) 7 (Fig. 1 F), vimentin (Fig. 1 G), and P63 (Fig. 1 H) and focally positive for epithelial membrane antigen (EMA). The nodule located in the RLL measured 3 cm × 2.5 cm, with multiple calcification. Pathological examination showed that the nodule consisted mainly of a solid component (Fig. 2 E), a mixed sclerotic component (Fig. 2 F), and a hemorrhagic hemangiomatous component (Fig. 2 G), with positive expression of thyroid transcription factor-1 (TTF-1) (Fig. 2 H), vimentin (Fig. 2 I), and EMA (Fig. 2 J), and negative expression of CD34, pan cytokeratin (PCK), and CD99. Pathologic examination proved that the mass in RUL was PSCC, a rare type of PSC, with pT_1_ N_0_ M_0_ (Ib stage) staging. The mass in the RLL was a PSP. The patient did not experience any complications after surgery. No radiologic findings of recurrence were observed on follow-up CT performed within 7 months. Our study obtained ethical approval from our hospital (Ethics Committee of the Affiliated Hospital of Southwest Medical University).

**Figure 1:**
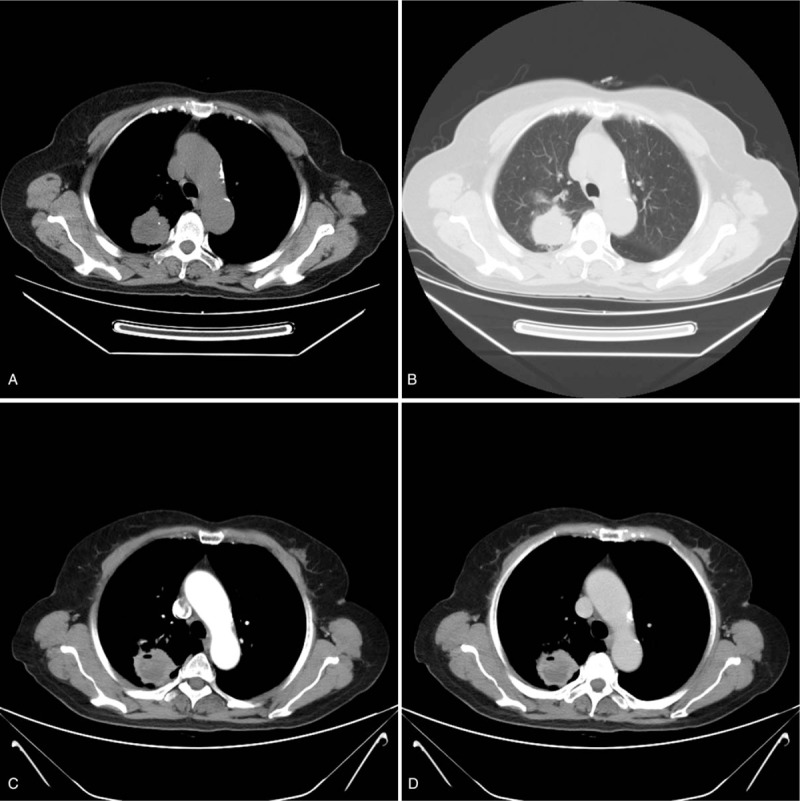
(A) PSCC in the RUL of the lung. CT shows a pulmonary mass located separately in RUL with a central low-attenuation area and a calcification spot. (B) CT image obtained using lung window settings showing peritumoral areas of ground-glass attenuation. (C and D) Contrast-enhanced CT performed after percutaneous needle biopsy, showing inhomogeneous enhancing mass lesion with a central low-attenuation area and an intratumoral cavity. (E) The pulmonary mass specimen demonstrates an abundance of spindle-shaped cancer cells (hematoxylin and eosin stain, ×200). (F–H) Immunohistochemical staining of the mass in the RUL is positive for several mesenchymal markers, including CK (F, ×200), vimentin (G, ×200), and p63 (H, ×200). CT = computer tomography, PSCC = pulmonary spindle cell carcinoma, RLL = the right lower lobe.

**Figure 1 (Continued):**
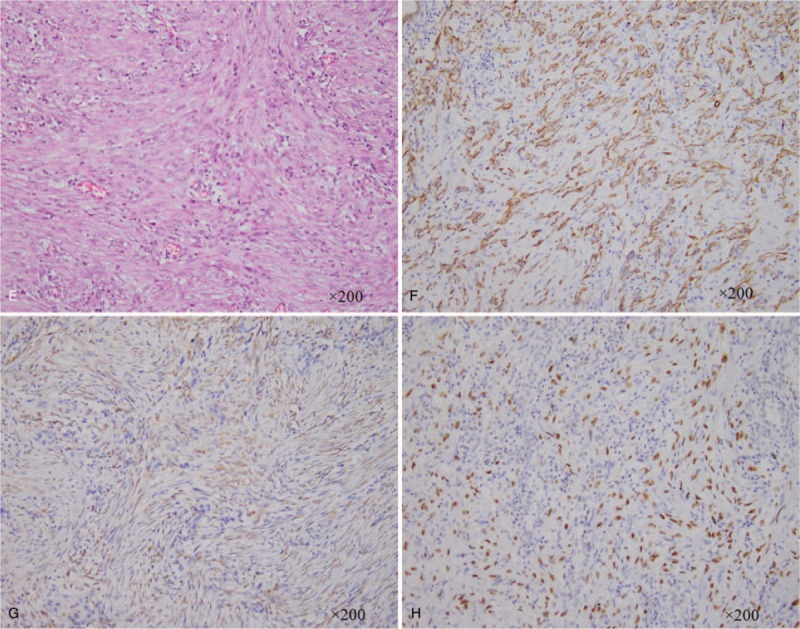
(A) PSCC in the RUL of the lung. CT shows a pulmonary mass located separately in RUL with a central low-attenuation area and a calcification spot. (B) CT image obtained using lung window settings showing peritumoral areas of ground-glass attenuation. (C and D) Contrast-enhanced CT performed after percutaneous needle biopsy, showing inhomogeneous enhancing mass lesion with a central low-attenuation area and an intratumoral cavity. (E) The pulmonary mass specimen demonstrates an abundance of spindle-shaped cancer cells (hematoxylin and eosin stain, ×200). (F–H) Immunohistochemical staining of the mass in the RUL is positive for several mesenchymal markers, including CK (F, ×200), vimentin (G, ×200), and p63 (H, ×200). CT = computer tomography, PSCC = pulmonary spindle cell carcinoma, RLL = the right lower lobe.

**Figure 2:**
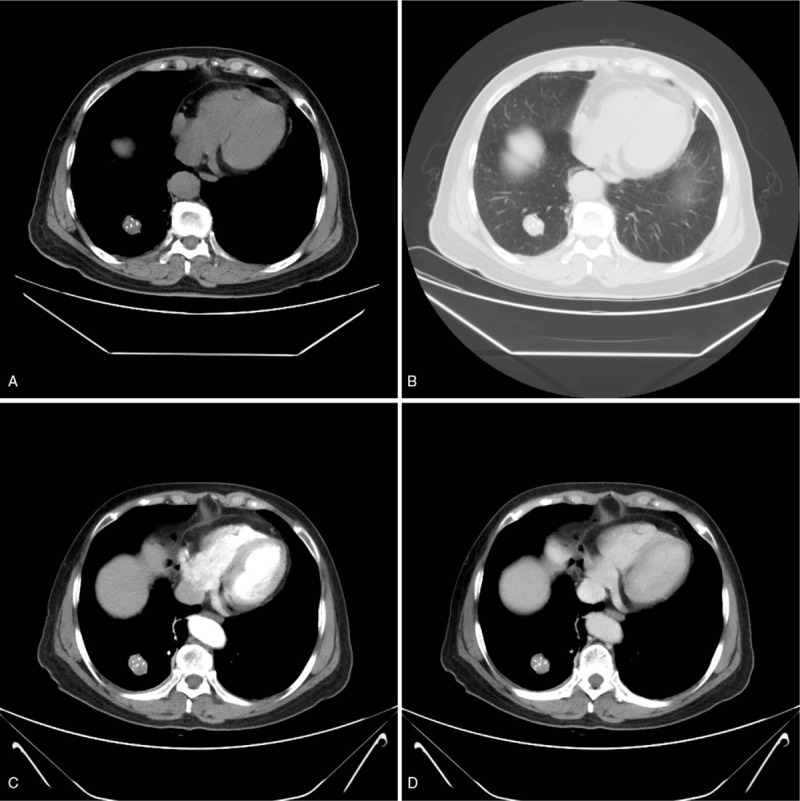
PSP in the RLL of the lung. CT shows a well-defined, round, peripheral mass with multiple nodular calcifications located in the RLL. (A) CT image obtained using lung window settings shows a tumor with smooth margins. (B) Contrast-enhanced CT shows homogeneous and height enhancement. (C and D) (E–J) Histopathological examination of PSP showing solid areas (E, ×200), sclerotic areas (F, ×200), and hemorrhagic areas (G, ×200). Immunohistochemical staining showing that the tumor was positive for TTF-1 (H, ×400), vimentin (I, ×400), and EMA (J, ×200).

**Figure 2 (Continued):**
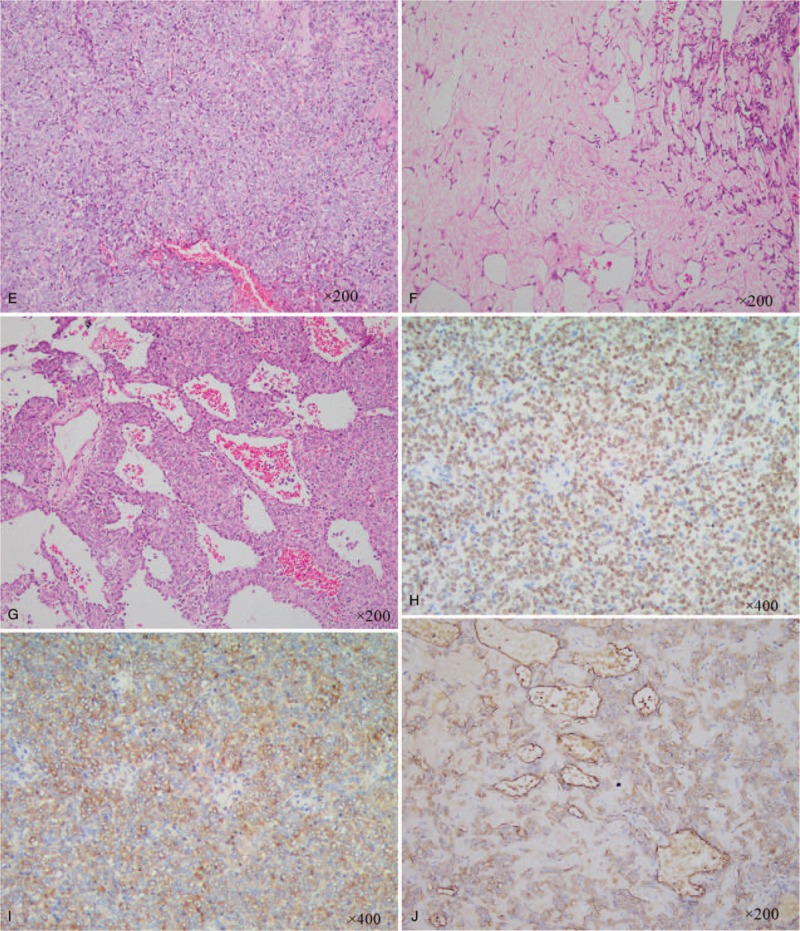
PSP in the RLL of the lung. CT shows a well-defined, round, peripheral mass with multiple nodular calcifications located in the RLL. (A) CT image obtained using lung window settings shows a tumor with smooth margins. (B) Contrast-enhanced CT shows homogeneous and height enhancement. (C and D) (E–J) Histopathological examination of PSP showing solid areas (E, ×200), sclerotic areas (F, ×200), and hemorrhagic areas (G, ×200). Immunohistochemical staining showing that the tumor was positive for TTF-1 (H, ×400), vimentin (I, ×400), and EMA (J, ×200).

## 3 Discussion

PSC is a rare subtype of lung carcinoma. It is aggressive and has a complex differential diagnosis. It mainly occurs in men aged over 60 years and in smokers. It usually presents with non-specific symptoms, including chest discomfort, chest pain, and coughing. Patients have a median survival of 11.54 months. The 1-year survival rate is 32%, and the 2, 3, and 5-year survival rates are 30%, 25%, and 21%, respectively.^[3]^ The following are the 5 subtypes of PSC, according to the 2015 WHO classification: pleomorphic carcinoma, spindle cell carcinoma, giant cell carcinoma, carcinosarcoma, and pulmonary blastoma.^[3]^ Pathological examination in our case was indicative of spindle cell carcinoma, which is an uncommon subtype of PSC. However, these histological subtypes appear to respond similarly to treatment.^[6]^

There is no effective treatment for PSC, owing to its pleiomorphic nature and heterogeneity. Surgical resection is effective and provides adequate local control for early-stage PSC. Platinum-based chemotherapy is commonly used for treatment, although its efficacy is not ideal. Significant advances have been made in targeted molecular therapy for the treatment of molecularly defined subsets of NSCLC. The frequency of EGFR mutations in PSC is controversial,^[7]^ but early diagnosis can increase the survival rate. A few studies have reported the CT features of PSC. It is mostly located peripherally in the right upper and left upper lobes, with a central low-attenuation area within the tumor. The tumor is massive and often invades the chest wall or pleura.^[8]^ Furthermore, peritumoral areas of ground-glass attenuation are a characteristic of specific subtypes, as observed in our patient.^[9]^ A calcification spot was also found in this patient. The existence of any significant correlation between calcification and spindle cell carcinoma is currently unclear. Most of the tumors display heterogeneous enhancement on contrast-enhanced CT, and the irregular areas correspond to the low-attenuation lesions within the tumors. A study demonstrated that a large low-density area within the tumor should be recognized as a CT feature, which is suggestive of a poorer prognosis.^[10]^ The clinical and CT features of pulmonary spindle cell carcinoma are unclear because of its rarity, and the accumulation of case reports is important. Our patient was a woman, without a history of smoking. Besides this, there are no special clinical or CT features that distinguish it from other types of PSC.

PSP is an uncommon benign epithelial tumor originating from type II pneumocytes. Microscopically, it is composed of cuboidal surface cells and round stromal cells. PSP exhibits 4 growth patterns, including papillary, solid, sclerotic, and hemorrhagic patterns. Most tumors contain at least 3 of the 4 growth patterns. They express CK7, TTF-1, and EMA on immunohistochemical analysis. PSP behaves in a clinically benign fashion, and simple enucleation or resection is sufficient.^[11]^

The majority of radiological studies reported are case studies or small-sample research projects. The value of preoperative radiological diagnosis of PSP is also a confusing issue. PSP has some CT characteristics, including well-defined round or ovoid masses located at the periphery or in the hilum, smooth margins, homogeneous attenuation, and strong enhancement.^[12]^ It also has a variety of uncommon features, including the air crescent sign, a prominent pulmonary artery, halo sign, and stippled calcification.^[13]^ In our case, the mass showed multiple nodular calcifications, which is a rare feature of PSP. To the best of our knowledge, calcification might be present in a few cases.^[14]^ Considering that the lesion was first discovered over 20 years ago, we can only speculate if multiple calcifications are associated with the duration of the tumor's growth. Unfortunately, we do not know if any CT images were obtained 20 years ago. The dynamic characteristics of PSP depend on the predominance of the hemangiomatous or papillary component (early and strong enhancement component) and solid component or sclerotic component (slow and persistent enhancement and little washout).^[15]^ Enhanced heterogeneity and homogeneity may be determined by the average diameters of the tumors. Pathological examination demonstrated the presence of 3 patterns in our case, and the lesion showed rapid, persistent, and homogeneous enhancement. A few cases of PSP may manifest as multiple tumors, which are often located within the same lobe. Although PSP is a benign tumor, it can also occur in the hilar or mediastinal nodes or undergo lung-to-lung metastasis. However, neither nodal, nor pulmonary metastasis was found in the present case. In conclusion, we reported a case of PSCC in a woman with PSP with multiple calcification. We observed the exceptional concomitance of these 2 extremely rare conditions. This case report fully reflects the importance of the differential diagnosis of benign and malignant solitary pulmonary nodules and highlights the fact that a typical imaging feature of lung abscess may also be associated with a rare and aggressive malignant tumor, which requires careful consideration and treatment. We found that the imaging features of PSCC in our patient were similar to those of other types of PSC. However, this aspect requires further research because of the rarity of PSCC. It also leads us to consider whether multiple calcification of PSP is associated with the duration of tumor growth. Nonetheless, the low prevalence of both diseases has made it difficult to explore the depth of their association.

## Author contributions

**Supervision:** Shu Jian.

**Writing – original draft:** Xu Lulu.

Shu Jian orcid: 0000-0002-7386-7217.

Xu Lulu orcid: 0000-0002-9971-9474.
